# Esketamine prevents propofol-induced injection pain: Randomized controlled trial

**DOI:** 10.3389/fphar.2022.991559

**Published:** 2022-09-20

**Authors:** Chaozhi Xu, Xiaotang Wei, Cuiwen Zhang, Xiaofang Huang, Hongmeng Lan, Yanping Xu, Xiaoyan Wu, Fuping Li, Xuehai Guan

**Affiliations:** ^1^ Department of Anesthesiology, The First Affiliated Hospital of Guangxi Medical University, Nanning, China; ^2^ Department of Anesthesiology, The People`s Hospital of Baise, Base, China; ^3^ Department of Anesthesiology, The Second People`s Hospital of Qinzhou, Qinzhou, China

**Keywords:** esketamine, propofol, general anesthesia, propofol-induced injection pain, propofol-induced pain, clinical trial

## Abstract

**Background:** Propofol is widely used during anesthesia. However, propofol-induced injection pain (PIP) is considered an unpleasant perioperative outcome. This study aimed to investigate the efficacy of a mixture of esketamine and propofol in preventing propofol injection pain in patients undergoing general anesthesia.

**Methods:** This was a prospective, double-blind, multicenter, and randomized controlled trial. We included 252 adult patients with the American Society of Anesthesiologists physical status I to II who underwent surgery under general anesthesia. Patients were randomly allocated in a 1:1:1:1 ratio to four groups (n = 63 per group). Group NS received a mixture of 1% propofol (20 ml) and 0.9% normal saline (1 ml), group ESK-4 received a mixture of 1% propofol (20 ml) and esketamine 4 mg (diluted with 0.9% normal saline, 1 ml), group ESK-12 received a mixture of 1% propofol (20 ml) and esketamine 12 mg (diluted with 0.9% normal saline, 1 ml), and group ESK-20 received a mixture of 1% propofol (20 ml) and esketamine 20 mg (diluted with 0.9% normal saline, 1 ml) as sedative drugs during anesthesia. The primary outcome was the incidence and distribution of different degrees of PIP. The secondary outcomes were vital signs, characteristics of surgery and anesthesia, and adverse events.

**Results:** The incidence of PIP in group ESK-20 (33.3%) was significantly lower than that in groups NS, ESK-4, and ESK-12 (63.3%, 62.2%, and 49.1%, respectively; *p* < 0.01). The incidence of moderate PIP in group NS (33.3%) and group ESK-4 (22.6%) was higher than that in groups ESK-12 (7.5%) and ESK-20 (6.7%). The incidence of severe PIP in group NS (6.7%) and group ESK-4 (9.4%) was higher than that in groups ESK-12 (1.9%) and ESK-20 (0%). There were no differences in the vital signs, characteristics of surgery and anesthesia, or adverse events between the groups.

**Conclusion:** Our results indicated that the esketamine–propofol admixture reduced the incidence of PIP in patients undergoing general anesthesia without severe side effects.

## 1 Introduction

Propofol is the most widely used anesthetic drug for induction and maintenance of general anesthesia. Although propofol is an ideal anesthetic agent owing to its rapid onset and offset, the incidence of propofol-induced injection pain (PIP) has varied from 28% to 90% (Canbay et al., 2008). PIP is considered an unpleasant encounter in anesthesia practice (Jalota et al., 2011). Many techniques have been developed to reduce the incidence of PIP, including changing the temperature and concentration of propofol (Jeong and Yoon, 2016; Lu et al., 2021), controlling the injection speed, selecting large vein vessels (Canbay et al., 2008; Desousa, 2016), and transcutaneous electrical acupoint stimulation (Jin et al., 2022). The most common techniques are pre-treatment or mixed use of propofol with drugs such as lidocaine (Euasobhon et al., 2016; Hong et al., 2016; Jeong and Yoon, 2016; Sun et al., 2016; Zirak et al., 2016; Xing et al., 2018; Tian et al., 2021; Wasinwong et al., 2022), nonsteroidal anti-inflammatory drugs (Madan et al., 2016; Miniksar, 2022), dexmedetomidine (Yu et al., 2015; Lu et al., 2021), ketamine (Cheng et al., 2017; Akbari et al., 2018), nitrous oxide (Kaabachi et al., 2007), opioids (Kizilcik et al., 2015; Lee et al., 2016; Singh et al., 2016; Lee et al., 2017; Wang et al., 2020), benzodiazepines (Guan et al., 2021), and magnesium sulfate (Sun et al., 2016). All of these techniques or drugs attenuated PIP to varying degrees, but their drawbacks, such as laryngospasm (Batra et al., 2005; Kaabachi et al., 2007), emergence agitation (Kaabachi et al., 2007), gastrointestinal ulcer (Madan et al., 2016), pulmonary embolism (Davies et al., 2002), lengthy onset (Wang et al., 2020), and tinnitus or dizziness (Xing et al., 2018), limited their clinical use, and PIP could not be completely abolished.

Ketamine is an N-methyl-d-aspartate receptor (NMDAR) antagonist with analgesic effects. Appropriate use of ketamine reduces postoperative pain (Himmelseher and Durieux, 2005). Pre-treatment or mixed use with ketamine reduced the incidence of PIP by approximately 30% in pediatric patients (Barbi et al., 2003; Batra et al., 2005; Cheng et al., 2017; Akbari et al., 2018). Currently, ketamine is a racemic mixture of levo-ketamine and right-ketamine (esketamine). However, psychotropic adverse effects limit the clinical use of ketamine (Batra et al., 2005). The affinity for NMDAR and the analgesic effect of esketamine are twice those of ketamine and induce fewer adverse events. However, the efficacy of esketamine in relieving PIP remains unclear. We hypothesized that a mixture of esketamine and propofol would reduce the incidence of PIP in patients undergoing general anesthesia.

## 2 Methods

### 2.1 Study design and patients

This was a prospective, double-blind, multicenter, and randomized controlled trial. This study was approved by the Medical Ethics Committee of the First Affiliated Hospital of Guangxi Medical University (identifier:2021-KY-E-138), and written informed consent was obtained from all participants. The study was performed at the First Affiliated Hospital of Guangxi Medical University, People’s Hospital of Baise, and Second People’s Hospital of Qinzhou. The trial was registered before patient enrollment (https://www.chictr.org.cn/showproj.aspx?proj=129317). This trial was performed in accordance with the Declaration of Helsinki and adhered to the 2010 CONSORT statement.

252 subjects (aged 18–60 years, the American Society of Anesthesiologists [ASA] physical status I–II, competent to provide informed consent) undergoing elective surgery under general anesthesia were enrolled. Exclusion criteria included a history of liver and kidney insufficiency, poor respiratory function, allergy to the drugs studied, severe hypertension, intracranial pressure, mental disorders, hyperthyroidism without treatment, or insufficient treatment. Patients taking sedatives or analgesics and vulnerable groups (infants, pregnant women, and elderly patients) were also excluded.

### 2.2 Randomization and masking

The randomization schedule was generated by EpiCalc 2000 software. Patients were randomly allocated in a 1:1:1:1 ratio to four groups (n = 63 per group). Different doses of esketamine were used in this study. Group NS received a mixture of 1% propofol (20 ml; Guangdong JiaBo Pharmaceutical Co., China) and 0.9% normal saline (NS, 1 ml), group ESK-4 received a mixture of 1% propofol (20 ml) and esketamine (ESK) 4 mg (1 ml, diluted with 0.9% NS; Jiangsu Hengrui Medicine Co., China), group ESK-12 received a mixture of 1% propofol (20 ml) and esketamine 12 mg (diluted with 0.9% NS, 1 ml), and ESK-20 received a mixture of 1% propofol (20 ml) and esketamine 20 mg (diluted with 0.9% NS, 1 ml) as sedative drugs during the anesthesia. Patients, researchers, surgeons, data collectors, statistical analysts, and anesthetists performing anesthesia or in charge of the PACU were blinded to group allocation. Sealed envelopes were used for concealment of group assignments until an assistant who did not participate in anesthesia or research prepared the drugs.

### 2.3 Interventions

The patients received no premedication and fasted for 8 hours. Clear liquids were allowed up to 2 h before anesthesia. A 20-gauge cannula was inserted into the vein on the dorsum of the hand, and a three-way tap was directly connected to the catheter 15 min before anesthesia. An infusion of Ringer’s lactate (5 ml/kg/h) was initiated to maintain patency. Routine monitoring was conducted after arriving at the operating room, including pulse oxygen saturation (SpO_2_), electrocardiogram, and noninvasive blood pressure. Continuous EEG monitoring was performed using a bispectral index (BIS) monitor (Covidien, United States) with four electrodes placed on the patient’s forehead. The infusion of Ringer’s lactate was stopped during the induction phase. Propofol and esketamine were mixed several minutes before induction. General anesthesia was induced with intravenous injection of a mixture of 1% propofol (20 ml) and 0.9% NS (1 ml) in the group NS, a mixture of 1% propofol (20 ml) and esketamine 4 mg (diluted with 0.9% NS, 1 ml) in the group ESK-4, a mixture of 1% propofol (20 ml) and esketamine 12 mg (diluted with 0.9% NS, 1 ml) in the group ESK-12, and a mixture of 1% propofol (20 ml) and esketamine 20 mg (diluted with 0.9% NS, 1 ml) in the group ESK-20 as sedative drugs at 10 ml/min using an electronic syringe pump until BIS reached the range of 40–60. After loss of consciousness (LOC, unresponsive to shaking shoulder), cisatracurium (0.3 mg/kg IV; Sinopharm Chemical Reagent Co., China) and fentanyl (3ug/kg IV; Yichang Humanwell Pharmaceutical Co., China) were administered. Patients were intubated after muscle relaxation. After completion of intubation, the mixture of propofol and esketamine was stopped, and anesthesia was maintained with propofol and remifentanil (Yichang Humanwell Pharmaceutical Co., China) using a target-controlled infusion pump and regulated according to clinical signs (blood pressure, heart rate, tears, and sweating) and the BIS value (maintained between 40 and 60). Cisatracurium was administered as necessary. All patients were mechanically ventilated (respiratory rate, 12 breaths/min; tidal volume, 8 ml/kg; fresh air flow, 2 L/min; oxygen concentration, 60%). Vasoactive agents such as ephedrine and atropine were administered to maintain hemodynamic stability.

All surgeries were performed according to clinical practice. All drugs were discontinued after the surgery was completed. All patients were transferred to the post-anesthesia care unit (PACU) for recovery and extubation.

### 2.4 Outcome measures

The primary outcomes were incidence of PIP and distribution of pain to different degrees during the induction of anesthesia. An anesthetist performing the anesthesia was blinded to group allocation and was trained to use a four-point pain scale (Guan et al., 2021) in each center to evaluate the severity of PIP continually during induction for all patients. Briefly, grade 0 indicates no pain (negative response to questioning); grade 1, mild pain (pain reported in response to questioning, without behavioral signs); grade 2, moderate pain (pain reported in response to questioning and accompanied by a behavioral sign or pain reported spontaneously); and grade 3, severe pain (strong vocal response or response accompanied by facial grimacing, arm retraction, or tears).

The secondary outcomes included vital signs (blood pressure, heart rate, and SpO2) and BIS before anesthesia; at LOC; at 0, 1, and 5 min after endotracheal intubation; at the beginning of operation; at the end of the operation; at the time of eye-opening; at the time of extubation; and at the time of leaving the PACU. The following parameters were recorded: time from induction to LOC, time from anesthesia induction to BIS ≤60, time from drug withdrawal to eye-opening, time from drug withdrawal to extubation, and length of PACU stay and surgery. The consumption of analgesics, sedatives, muscle relaxants, and vasoactive drugs was recorded. The secondary outcomes also included the following adverse events from anesthesia induction to leaving PACU: hypertension (20% increase in mean arterial pressure from baseline), hypotension (20% decrease in mean blood pressure from baseline), bradycardia (<50 beats/min), tachycardia (>100 beats/min), delirium (an acute disturbance in attention and cognition) (Hshieh et al., 2018), dysphoria (an inner uneasiness or unfounded fear that lacks obvious objective reasons), nausea, and vomiting.

### 2.5 Sample size

Our preliminary study revealed that the incidence of PIP was approximately 50%–60% in our institutions. We hypothesized a 50% reduction in the incidence of PIP based on an alpha of 0.05 and a power of 80%. Under these assumptions, 52 patients were included in each group to detect any significant differences. Considering the potential loss (20%) to follow-up, we increased the sample size to 63 in each group.

### 2.6 Statistical methods

Data are presented as mean ± SD or number of cases (%). Statistical analyses were performed using GraphPad Prism 9.0 (GraphPad Software, San Diego, California United States). Continuous data were analyzed using one-way or two-way analysis of variance, where appropriate. Categorical data were analyzed using the Chi-square tests (when <20% cells have an expected count less than 5) or Fisher’s exact test (when ≥20% cells have an expected count less than 5, or at least one cell have an expected count less than 1. The significance level was set at *p* < 0.05.

## 3 Results

**FIGURE 1:**
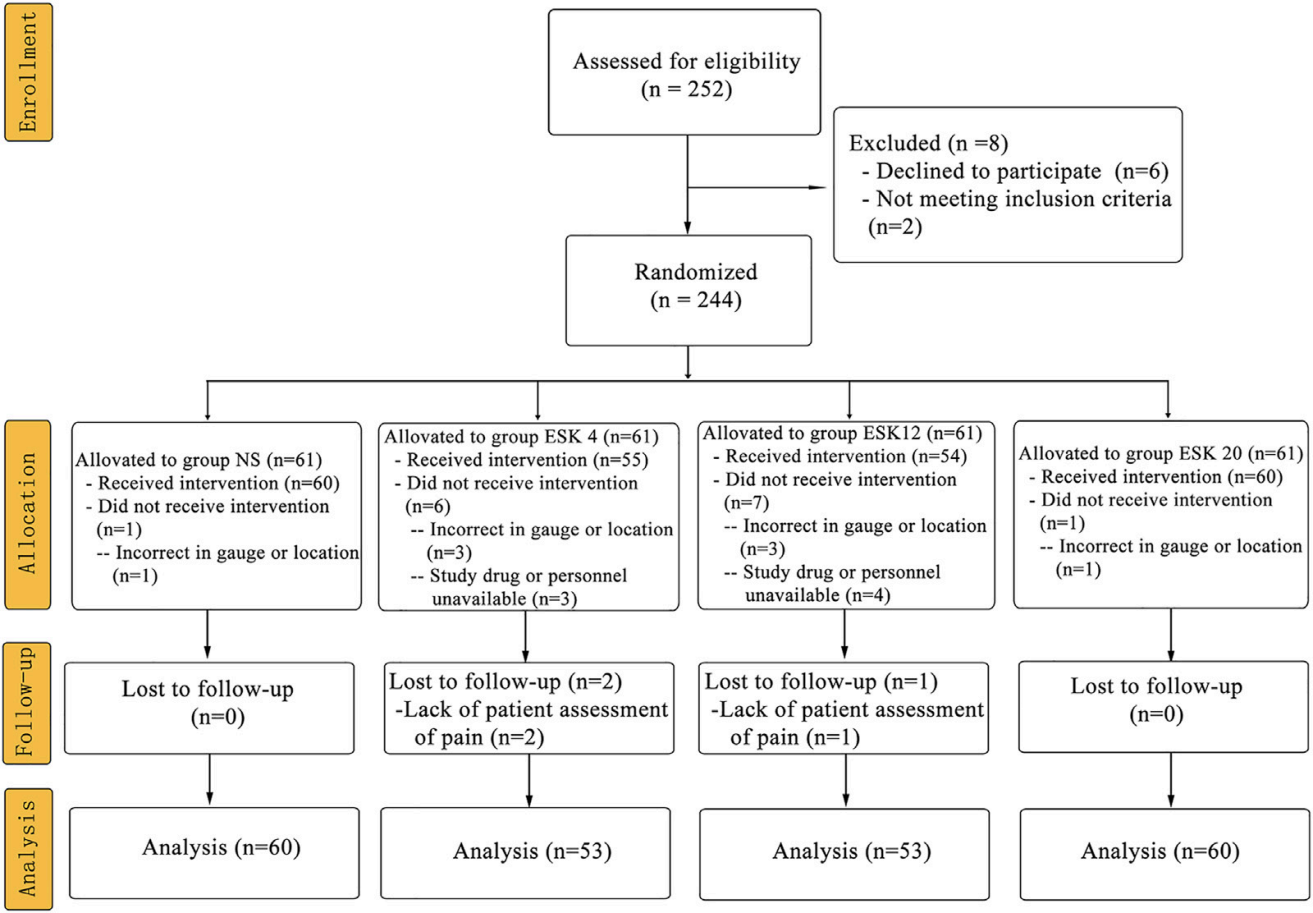
CONSORT flow of clinical procedures. Abbreviations: NS: normal saline; ESK: esketamine.

From July 5, 2021, to March 30, 2022, among 252 patients recruited for this study, six declined to participate, and two did not meet the inclusion criteria. A subset of 244 patients was randomly assigned to four groups, and 226 patients were analyzed (Figure 1). Overall, the demographic data were similar among the groups in terms of age, height, body weight, body mass index, male/female ratio, ASA score, and Mallampati score (Table 1).

**TABLE 1:** Patient demographic data.

Parameter	NS (n = 60)	ESK-4 (n = 53)	ESK-12 (n = 53)	ESK-20 (n = 60)	*p* value
Age (y)	42.13±11.60	41.08±11.62	42.73±10.57	41.61±11.45	0.743
Height (cm)	162.2±7.61	163.5±8.42	161.0±8.04	161.5±8.13	0.359
Body weight (kg)	59.15±11.14	63.83±9.82	60.26±9.54	60.98±11.04	0.113
BMI (kg/m^2^)	22.70±3.70	23.97±4.05	23.21±3.01	23.33±3.60	0.321
Male/female respondent	31/29	30/23	27/26	32/28	0.938
ASA score (I/II)	15/45	11/42	15/38	22/38	0.273
Mallampati score (I/II)	12/48	12/41	10/43	15/45	0.858

Data are displayed as the mean ± SD or number of cases. One-way analysis of variance was used to analyze the age, height, body weight, and BMI between the groups. Chi-squared tests were used to analyze sex, ASA, and Mallampati scores. No statistically significant differences were observed between the groups.

Abbreviations: NS, normal saline; ESK, esketamine; BMI, body mass index; ASA, American Society of Anesthesiologists.

The primary outcomes are presented in Table 2. The incidence of PIP was significantly lower in group ESK-20 (33.3%; *p* = 0.001, compared with NS; *p* = 0.002, compared with ESK-4) than in group NS (63.3%), group ESK-4 (62.2%), and group ESK-12 (49.1%). There were no significant differences between the ESK-12, ESK-4, and NS groups (*p* > 0.05). No significant difference was found in the incidence of mild PIP among the four groups (*p* > 0.05). The percentage of patients with moderate PIP was lower in group ESK-20 (6.7%; *p* < 0.0001, compared with NS; *p* = 0.015, compared with ESK-4) and group ESK-12 (7.5%, *p* = 0.001, compared with NS) than in group NS (33.3%) and group ESK-4 (22.6%). The percentage of patients with severe PIP was lower in group ESK-20 (0%; *p* = 0.119, compared with NS; *p* = 0.016, compared with ESK-4) and group ESK-12 (1.9%; *p* = 0.218, compared with NS; *p* = 0.093, compared with ESK-4) than in group NS (6.7%) and group ESK-4 (9.4%), although some of these differences do not reach statistical significance.

**TABLE 2:** Incidence of propofol–induced injection pain.

Parameter	NS (n = 60)	ESK-4 (n = 53	ESK-12 (n = 53)	ESK-20 (n = 60)	*p*-value	
					ESK-20 vs. NS	ESK-20 vs. ESK-4
Patients with pain [No. (%)]	38 (63.3%)	33 (62.2%)	26 (49.1%)	20 (33.3%)	0.001	0.002
The severity of pain [No. (%)]						
0	22 (36.7%)	20 (37.7%)	27 (50.9%)	40 (66.7%)	0.001	0.002
1	14 (23.3%)	16 (30.2%)	21 (39.6%)	16 (26.7%)	0.673	0.678
2	20 (33.3%)	12 (22.6%)	4 (7.5%)	4 (6.7%)	0.000	0.015
3	4 (6.7%)	5 (9.4%)	1 (1.9%)	0 (0%)	0.119	0.016

Data are presented as the number of cases. Chi-square tests (when <20% cells have an expected count less than 5) or Fisher’s exact test (when ≥20% cells have an expected count less than 5, or at least one cell has an expected count less than 1) was used to analyze the incidence of PIP.

Abbreviations: NS, normal saline; ESK, esketamine.

The characteristics of anesthesia and surgery are shown in Table 3. The time from induction to LOC in groups ESK-12 (82.0±31.1 s) and ESK-20 (89.3±35.2 s) was shorter than that in groups NS (98.7±35.4 s) and ESK-4 (95.3±35.7 s), but there were no significant differences. In addition, there were no significant differences in time from anesthesia induction to BIS ≤60, time from drug withdrawal to eye-opening, time from drug withdrawal to extubation, length of PACU stay and surgery, and distribution of surgery (*p* > 0.05). No significant differences were observed in the crystalloid infusion volume between the groups (*p* > 0.05).

**TABLE 3:** Characteristics of anesthesia and surgery.

Parameter	NS (n = 60)	ESK-4 (n = 53)	ESK-12 (n = 53)	ESK-20 (n = 60)
Time from induction to LOC (s)	98.7±35.4	95.3±35.7	82.0±31.1	89.3±35.2
Time from induction to BIS≤60 (s)	120.1±47.9	126.5±68.6	110.3±59.3	122.2±71.8
Length of anesthesia (min)	117.1±60.2	111.6±52.7	96.8±41.3	105.2±50.2
Time from drug withdrawal to eye-opening (min)	14.4±8.9	14.7±10.5	14.7±9.8	14.0±7.8
Time from drug withdrawal to extubation (min)	18.9±9.9	19.3±12.5	19.5±11.9	18.5±10.2
Length of PACU stay (min)	61.3±20.0	66.0±21.7	63.1±20.2	60.0±20.2
Length of surgery (min)	92.9±54.5	90.9±54.6	75.1±39.2	79.7±47.9
Infusion volume (ml)	782.5±413.8	713.6±268.9	733.1±268.6	700.8±359.6
Distribution of surgery				
Urology surgery [No. (%)]	32 (53.3%)	31 (58.5%)	32 (60.4%)	29 (48.3%)
General surgery [No. (%)]	20 (33.3%)	14 (26.4%)	11 (20.8%)	19 (31.7%)
Gynecology surgery [No. (%)]	5 (8.3%)	6 (11.3%)	6 (11.3%)	9 (15.0%)
Orthopedics surgery [No. (%)]	1 (1.7%)	2 (3.8%)	2 (3.8%)	1 (1.7%)
Otolaryngology surgery [No. (%)]	2 (3.3%)	0 (0%)	2 (3.8%)	2 (3.3%)

Data are displayed as the mean ± SD, or number of cases. One-way analysis of variance was used to analyze differences between groups. No statistically significant differences were observed between the groups.

Abbreviations: NS, normal saline; ESK, esketamine; LOC, loss of consciousness; BIS, bispectral index; PACU, post-anesthesia care unit.

The consumption of anesthetic and vasoactive drugs is shown in Table 4. Sedative use was recorded at different time points. There were no differences in the overall consumption of propofol, fentanyl, remifentanil, cisatracurium, or ephedrine among the four groups (*p* > 0.05).

**TABLE 4:** Consumption of anesthetic and vasoactive drugs.

Parameter	NS (n = 60)	ESK-4 (n = 53)	ESK-12 (n = 53)	ESK-20 (n = 60)
Total consumption of pro (mg)	767.5±418.5	749.0±422.5	679.7±269.8	773.2±358.1
LOC	109.3±29.9	115.4±35.1	110.2±27.4	105.9±24.8
BIS≤60	123.5±34.1	132.8±35.7	118.6±31.9	117.6±31.3
Intubation	152.7±33.2	159.8±30.5	148.0±30.0	150.2±31.8
Total consumption of ESK (mg)	0±0	3.2±0.6	8.9±1.8	14.9±3.1
Total consumption of remifentanil (mg)	0.78±0.48	0.81±0.51	0.68±0.37	0.74±0.42
Total consumption of fentanyl (mg)	0.22±0.05	0.22±0.05	0.21±0.04	0.20±0.05
Total consumption of cisatracurium (mg)	16.5±5.47	19.1±4.18	16.9±3.98	15.9±4.37
Total consumption of ephedrine (mg)	6.12±6.71	4.21±6.91	4.18±4.91	5.22±6.55
Consumption of ephedrine during induction (mg)	1.30±4.26	0.75±3.31	1.13±3.08	0.75±2.58
Consumption of ephedrine during maintenance (mg)	4.83±5.35	2.70±5.43	3.05±3.83	3.13±4.69

Data are displayed as mean ± SD., One-way analysis of variance was used to analyze differences between groups. No statistically significant differences were observed between the groups.

Abbreviations: NS, normal saline; ESK, esketamine; Pro, propofol; LOC, loss of consciousness; BIS, bispectral index.

**FIGURE 2:**
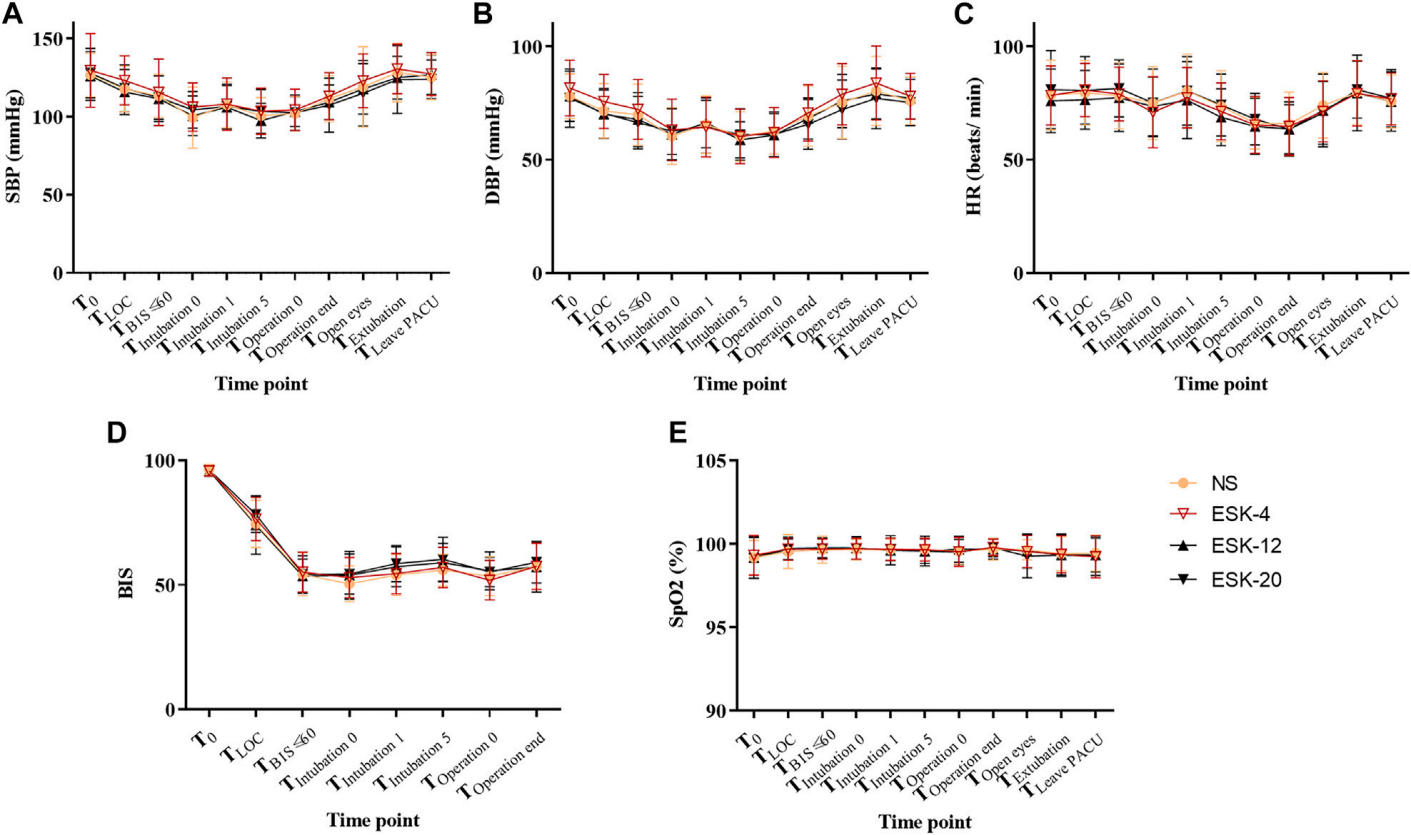
Changes in vital signs of patients. **A–E**: No statistically significant differences were observed between the groups. Abbreviations: SBP, systolic blood pressure; DBP, diastolic blood pressure; HR, heart rate; SPO_2_, pulse oximetry; NS, normal saline; ESK, esketamine; BIS, bispectral index.

No significant differences were found in systolic blood pressure, diastolic blood pressure, heart rate, SpO_2_, or BIS values at any time point between the four groups (Figure 2; *p* > 0.05).

All adverse events were classified as mild or moderate. No serious adverse events or discontinuation due to adverse events were reported in any group (Table 5). There were no significant differences between the groups in terms of the incidence of hypotension, bradycardia, dysphoria, nausea, or vomiting (*p* > 0.05). None of the patients developed hypertension, tachycardia, or delirium in any of the groups.

**TABLE 5:** Incidence of adverse events between the groups.

Parameter	NS (n = 60)	ESK-4 (n = 53)	ESK-12 (n = 53)	ESK-20 (n = 60)
Hypertension [No. (%)]	0 (0%)	0 (0%)	0 (0%)	0 (0%)
Total hypotension [No. (%)]	39 (65.0%)	25 (47.2%)	30 (56.6%)	30 (50.0%)
Hypotension during induction [No. (%)]	15 (25.0%)	9 (17.0%)	10 (18.9%)	9 (15.0%)
Hypotension during maintenance [No. (%)]	35 (58.3%)	21 (39.6%)	25 (47.2%)	25 (41.7%)
Tachycardia (>100 beats/min) [No. (%)]	0 (0%)	0 (0%)	0 (0%)	0 (0%)
Total bradycardia (<50 beats/min) [No. (%)]	31 (51.7%)	21 (39.6%)	20 (37.7%)	26 (43.3%)
Bradycardia during induction [No. (%)]	6 (10.0%)	2 (3.8%)	3 (5.7%)	3 (5.0%)
Bradycardia during maintenance [No. (%)]	25 (41.7%)	19 (35.8%)	17 (32.1%)	23 (38.3%)
Delirium [No. (%)]	0 (0%)	0 (0%)	0 (0%)	0 (0%)
Dysphoria [No. (%)]	1 (1.7%)	4 (7.5%)	0 (0%)	0 (0%)
Nausea/vomiting [No. (%)]	0 (0%)	0 (0%)	0 (0%)	1 (1.7%)

Data are displayed as the number of cases (%). Chi-square tests (when <20% cells have an expected count less than 5) or Fisher’s exact test (when ≥20% cells have an expected count less than 5, or at least one cell has an expected count less than 1) was used to analyze the incidence of adverse events. No statistically significant differences were observed between the groups.

Abrreviations: NS, normal saline; ESK, esketamine.

## 4 Discussion

To our knowledge, this study is perhaps the first to evaluate the efficacy of a mixture of esketamine and propofol in preventing PIP. Our results indicated that a mixture of esketamine (20 mg, 1 ml) and propofol (200 mg, 20 ml) dose significantly reduced the incidence of PIP in patients undergoing general anesthesia without severe side effects. There were no differences in the vital signs, characteristics of surgery and anesthesia, or adverse events between the groups.

However, the mechanism underlying PIP remains unclear. The primary mechanism is the direct irritation of afferent peripheral nerve fibers in the inner wall of the venous vessel. Peripheral nerve fibers sense nociceptive information and transfer it onward. Based on this mechanism, many techniques have been developed to reduce the incidence of PIP, such as changing the temperature and concentration of propofol (Jeong and Yoon, 2016; Lu et al., 2021), controlling injection speed, selecting large vein vessels (Canbay et al., 2008; Desousa, 2016), transcutaneous electrical acupoint stimulation (Jin et al., 2022), or pre-treatment or mixed use propofol with drugs, such as lidocaine (Euasobhon et al., 2016; Hong et al., 2016; Jeong and Yoon, 2016; Sun et al., 2016; Zirak et al., 2016; Xing et al., 2018; Tian et al., 2021; Wasinwong et al., 2022), nonsteroidal anti-inflammatory drugs (Madan et al., 2016; Miniksar, 2022), and dexmedetomidine (Yu et al., 2015; Lu et al., 2021).

The analgesic mechanism of N-methyl-d-aspartate (NMDA) receptor antagonists remains unclear. NMDA receptors are expressed in the primary afferent unmyelinated terminals that innervate the peripheral skin (Wang et al., 2000). Subcutaneous or intra-articular injection of NMDA receptor antagonists, such as ketamine, produced potent analgesia (Bai and Zhao, 1997; Lawand et al., 1997). These findings provide evidence of the potential role of peripheral NMDA in nociceptive transmission (Wong et al., 2014; Alhilou et al., 2021). Ketamine has been shown to have a local anesthetic action or additive hypnotic effect by acting on NDMA receptors in the vascular endothelium or central nervous system, respectively, when administered intravenously. For example, pretreatment with ketamine was effective in preventing PIP in children (Barbi et al., 2003; Tobias, 2004; Cheng et al., 2017) and adults (Tan et al., 1998; Suzuki et al., 2002; Zahedi et al., 2009; Wang et al., 2013; Thukral et al., 2015; Akbari et al., 2018). A propofol–ketamine mixture was more effective than ketamine pre-treatment in preventing PIP (Hwang et al., 2009). However, PIP was twice as high with a propofol–ketamine mixture than with a propofol–lidocaine mixture in children (Kaabachi et al., 2007). Moreover, the clinical use of ketamine is limited owing to its drawbacks, such as psychotomimetic/dissociative effects and abuse potential (Batra et al., 2005). Currently, ketamine is a racemic mixture of levo-ketamine and right-ketamine (esketamine). The affinity for NMDAR and the analgesic effect of esketamine are twice those of ketamine and induce fewer adverse events. In our study, the propofol–esketamine mixture (ESK-20 group) was effective in preventing PIP (decreased from 63.3% to 33.3%), and no patient reported severe pain. Pretreatment with esketamine (0.15–0.25 mg/kg) before injection of propofol significantly reduced the incidence of PIP (Li et al., 2022a; Fu et al., 2022). The mechanism by which esketamine reduces PIP may be related to its action on the peripheral NMDA receptors. The pH of the propofol–ketamine mixture was 5.84, while propofol had a pH of 7.86, supporting that pH changes are a more important cause of PIP (Hwang et al., 2009). We did not determine the pH of the propofol–esketamine mixture, and the change in pH may support our results. Esketamine is also a potent central-acting analgesic. The mechanism through which esketamine reduces PIP may be related to its central action too.

Mixing propofol with esketamine facilitated a simple and rapid injection sequence during anesthesia (Zheng et al., 2022). However, mixing them together may increase the risk of drug reactions. In the pilot study, no color change or immiscible surface layer was detected by visual inspection after mixing propofol with esketamine. In our study, we did not observe any adverse events after the use of the propofol–esketamine mixture.

The time from induction to LOC in groups ESK-12 and ESK-20 was shorter than that in groups NS and ESK-4, and the time from anesthesia induction to a BIS ≤60 was shorter in group ESK-12, although none of these effects reached statistical significance (*p* > 0.05). The consumption of propofol during the period of LOC and BIS ≤60 showed a trend toward lower values in group ESK-12 and group ESK-20, but the differences did not reach statistical significance (*p* > 0.05). The combined use of esketamine with propofol had no effect on the duration of anesthesia, did not reduce the total consumption of anesthetic drugs and vasoactive drugs, and had no effect on vital signs, which was inconsistent with previous studies in children (Zheng et al., 2022) and adults (Zhang et al., 2022). The interpretation may be that the doses we used were small, and different drugs such as midazolam were used in the other study (Zhang et al., 2022).

Emergence delirium or hallucinations are common drawbacks of esketamine or ketamine if either is used as the sole agent for sedation at higher doses (Zheng et al., 2022; Zhornitsky et al., 2022). Most psychiatric disorders associated with racemic ketamine come from (R)-ketamine (Tan et al., 2020), but not (S)-ketamine. Esketamine, the S-enantiomer of racemic ketamine, was approved for treatment of treatment-resistant depression in 2019. A lower dose of esketamine reduced these side events (Bornemann-Cimenti et al., 2016). No case of delirium was reported in our study; the combination of esketamine with propofol may have also attenuated this drawback (Hwang et al., 2009). Propofol has direct anti-emetic properties (Borgeat et al., 1992). Only one case of nausea/vomiting was reported in our study. Few patients in our study developed dysphoria. None of the patients had tachycardia or hypertension. No difference was found in the incidence of hypotension, bradycardia, dysphoria, nausea, or vomiting between the groups (Table 5). However, some studies revealed that 0.2 mg/kg esketamine for elderly subjects could maintain the stability of hemodynamics (Li et al., 2022b). This inconsistency may result in different uses of anesthetics such as etomidate, sufentanil, and inhaled anesthetics.

This study has several limitations. First, the inclusion of participants weakened the external validity of the trial. The study included only adult patients who underwent elective surgery. Therefore, our results may not be generalizable to other populations. Second, the number of participants in this study was relatively small. In the future, we will evaluate the efficacy of a mixture of propofol and esketamine in preventing PIP in children.

## 5 Conclusion

In conclusion, our current findings indicate that esketamine (20 mg) and 1% propofol (20 ml) admixture significantly reduced the incidence of PIP in patients undergoing general anesthesia without severe side effects.

## Data Availability

The original contributions presented in the study are included in the article/Supplementary Material; further inquiries can be directed to the corresponding author.
